# Biological response of cancer cells to radiation treatment

**DOI:** 10.3389/fmolb.2014.00024

**Published:** 2014-11-17

**Authors:** Rajamanickam Baskar, Jiawen Dai, Nei Wenlong, Richard Yeo, Kheng-Wei Yeoh

**Affiliations:** Division of Cellular and Molecular Research, Department of Radiation Oncology, National Cancer CentreSingapore, Singapore

**Keywords:** cancer cells, radiation, direct DNA damage, bystander effect

## Abstract

Cancer is a class of diseases characterized by uncontrolled cell growth and has the ability to spread or metastasize throughout the body. In recent years, remarkable progress has been made toward the understanding of proposed hallmarks of cancer development, care, and treatment modalities. Radiation therapy or radiotherapy is an important and integral component of cancer management, mostly conferring a survival benefit. Radiation therapy destroys cancer by depositing high-energy radiation on the cancer tissues. Over the years, radiation therapy has been driven by constant technological advances and approximately 50% of all patients with localized malignant tumors are treated with radiation at some point in the course of their disease. In radiation oncology, research and development in the last three decades has led to considerable improvement in our understanding of the differential responses of normal and cancer cells. The biological effectiveness of radiation depends on the linear energy transfer (LET), total dose, number of fractions and radiosensitivity of the targeted cells or tissues. Radiation can either directly or indirectly (by producing free radicals) damages the genome of the cell. This has been challenged in recent years by a newly identified phenomenon known as radiation induced bystander effect (RIBE). In RIBE, the non-irradiated cells adjacent to or located far from the irradiated cells/tissues demonstrate similar responses to that of the directly irradiated cells. Understanding the cancer cell responses during the fractions or after the course of irradiation will lead to improvements in therapeutic efficacy and potentially, benefitting a significant proportion of cancer patients. In this review, the clinical implications of radiation induced direct and bystander effects on the cancer cell are discussed.

## Introduction

Cancer is a complex disease, which grow locally and also possesses the capacity to metastasize to different organs in the body. Cancer continues to be a major disease and the numbers of cancer cases are projected to be more than double worldwide in the next 20–40 years and surpass heart disease as the leading cause of death (Jemal et al., 2010; Thun et al., 2010). Moreover, management of cancer is a rising concern in an aging population and is increasingly important in the developing countries (Siegel et al., 2012). International Agency for Research on Cancer (IARC) has predicted that by 2030, ~26 million new cancer cases and 17 million cancer deaths will occur each year worldwide (IARC, 2010). That compares to 12.7 million new cancers and 7.6 million cancer death reported by GLOBOCAN 2008. Despite initial high response rates to the various treatment modalities and interventions, a large proportion of cancer patients suffered relapse in years or decades later (Karrison et al., 1999; Weckermann et al., 2001; Pfitzenmaier et al., 2006; Aguirre-Ghiso, 2007), resulting a therapeutic challenge. Radiation therapy aims to deliver the optimal isodose to the tumor volume while sparing the normal tissues. For years, radiation biologists have thought that the biological effects induced by ionizing radiation are the direct consequence of a radiation induced DNA damage and thereafter death of cancer cell. In a recent seminal study Martin et al. (2014) reported that the rapid breakdown of a tumor could cause a flood of cancerous material, including intact cells to enter the lymphatic flow and form tumors in the distanced organs, a possible mechanism of the formation of therapy related metastasis. Therefore, past 20 years have seen a major paradigm shift in radiation biology and enormous progress has been made to understand the biological and molecular determinants of cellular radiation responses.

In recent years, many treatment and management options for cancer exist with the primary ones including: surgery, chemotherapy, radiation therapy and palliative care. Radiation therapy or radiotherapy is a highly effective tool for the cancer treatment and also an important component of cancer management, conferring a survival and palliative benefits (Prise, 2006; Guadagnolo et al., 2013; Liauw et al., 2013). In patients with inoperable tumors, radiation therapy is the only option (Durante and Loeffler, 2010). Furthermore, patients who are incompletely resected or recurrent of tumors after surgery are mostly treated by radiation therapy (Durante and Loeffler, 2010). Approximately 50% of all cancer patients receive radiation therapy during their course of illness (Delaney et al., 2005; Begg et al., 2011) either for cure or as a palliative treatment to relieve the patients from symptoms such as pain caused by the cancer (Delaney et al., 2005), majority of patients are treated with the intent to cure (Barnett et al., 2009). Although tremendous progress has been made toward understanding the hallmarks of cancer development and treatment response, a need remains to improve the curative rate by targeting multiple molecular pathways that mediate the DNA damage response.

Radiation therapy destroys cancer by depositing high physical energy of radiations on the cancer cells. The first clinical use of radiation for the cancer treatment was recorded in late 19th century (Connell and Hellman, 2009), soon after Roentgen discovered X-rays in 1895 and the effectiveness of radiation that has been developed over the years showed a drastic beneficial effects (Bernier et al., 2004; Giap and Giap, 2012). Over the years, radiation therapy has been driven by constant technological advances (Thariat et al., 2013) with the understanding of various molecular mechanisms involved in the treatment sensitivity and resistance (Jacinto and Hall, 2003; Camphausen and Tofilon, 2004; Sabatini, 2006; Kuwahara et al., 2014). In radiation oncology, research and development in the last three decades has led to a considerable improvement in our understanding of radiation dose and the dose-volume responses. Ionizing radiation has been harnessed for over a century to treat patients with cancer largely based on the rationale that the rapidly proliferating cancer cells are more sensitive than normal cells for the DNA damage response. Recently, our understanding of radiation effects has been expanded widely in terms of the consequences of radiation-induced tumor cell death and various signaling pathways involved in sensitivity, resistance and further molecular sensors that modify the tumor response to radiation. Though high-energy photons (X-rays and gamma rays) are the most common radiation modalities used in the external beam treatment, protons provide dosimetric advantages compared with photons. In this review, we discuss about the biological response of rapidly proliferating cancer cells to the radiation treatment.

## Radiation and biological implications

Radiation remains as most widely utilized treatment modalities in the clinical management of cancer (Burnette and Weichselbaum, 2013; McGale et al., 2014). Patients with localized malignant tumors are treated with radiation at some point in the course of their disease (Bentzen, 2006; Durante and Loeffler, 2010; Baskar et al., 2012; Moding et al., 2013). Radiation therapy is applied in a course of multiple fractions over several weeks to reduce the normal cell toxicity (Bentzen, 2006), with an estimation of about 40% toward the curative treatment (Barnett et al., 2009). Furthermore, radiation therapy is a highly cost effective with a single modality treatment accounting about only 5% of the total cost of cancer care (Ringborg et al., 2003). Therefore, any improvement in the efficacy of radiation therapy will therefore benefit a large number of patients. Recent advances in radiation therapy have enabled the use of different types of radiation sources like photons and protons for a better cancer treatment efficacy. Radiation therapy uses low and high linear energy transfer (LET) radiations to efficiently kill the tumor cells while minimizing dose (biological effective) to normal tissues to prevent toxicity (Lawrence et al., 2008; Niemantsverdriet et al., 2012). LET is defined as measurement of the number of ionizations which radiation causes per unit distance as it traverses the living cells or tissue. X-rays, gamma rays and charged particles are the most types of radiation used for cancer treatment. In radiation oncology, radiations can be delivered by a machine outside the body (external beam radiation therapy) or irradiated through the radioactive material placed in the body near to cancer cells/tissue (internal radiation therapy, also called brachytherapy). On the other hand, systemic radiation therapy uses radioactive substances, such as radioactive iodine, that travel in the blood to kill the cancer cells.

A better understanding of biological effects of radiation will lead to efficient use and better protection. Biological effectiveness of radiation depends on the linear energy transfer (LET), total dose, fractionation rate and radiosensitivity of the targeted cells or tissues (Hall, 2007). Low LET radiations (X-rays, gamma rays and beta particles) deposit a relatively small quantity of energy. On the other hand, radiation particles either negatively charged (electrons), positively charged (protons, alpha rays, and other heavy ions) deposits more energy on the targeted areas called the Bragg peak and causes more biological effects than the low LET radiations. However, tumors have developed multiple strategies to resist radiation damage. The following (1) Tumor burden (2) Tumor microenvironment/hypoxia (3) Inherent or acquired treatment resistance and (4) Repopulations during the treatment are the major mechanisms involved in the treatment resistance (Seiwert et al., 2007). Ionizing radiation effectively kills human cells; over a period sufficiently high doses of radiation can sterilize any tumor and achieve nearly 100% of tumor control probability (TCP) (Thariat et al., 2013), either alone or in combination with surgery and chemotherapy. However, when using external-beam radiation healthy tissues are unavoidably exposed to radiation, which increases the normal tissue complication probability. Over the years, technological improvements in radiation therapy delivery have aimed to widen the therapeutic window while reducing the normal tissue impact and increase in target tissue (tumor) control (Durante and Loeffler, 2010; Loeffler and Durante, 2013), and the benefits will be three-fold: patient cure, organ preservation and cost-efficiency.

The overall outcome of radiation treatment is cell or tissue damage; if it is not repairable eventually kill the cells. Effectiveness of radiation therapy that have been developed over years showed an increase in the number of cancer survivors, but preventing or reducing late effects are a significant public health issue. Furthermore, increase in the number of cancer survivors has stimulated interest in the quality of life of cancer survivors. The situation is important among non-elderly adults. In particular, children are inherently more radiosensitive and have more remaining years of life during which radiation induced late effect in normal cells could manifest in their hyperproliferation (Allan and Travis, 2005). However, understanding the tumor biology and considerable technical advancement (e.g., proton therapy) over the last three decades provides the opportunity for better cancer treatment.

## Direct effects

**Figure 1:**
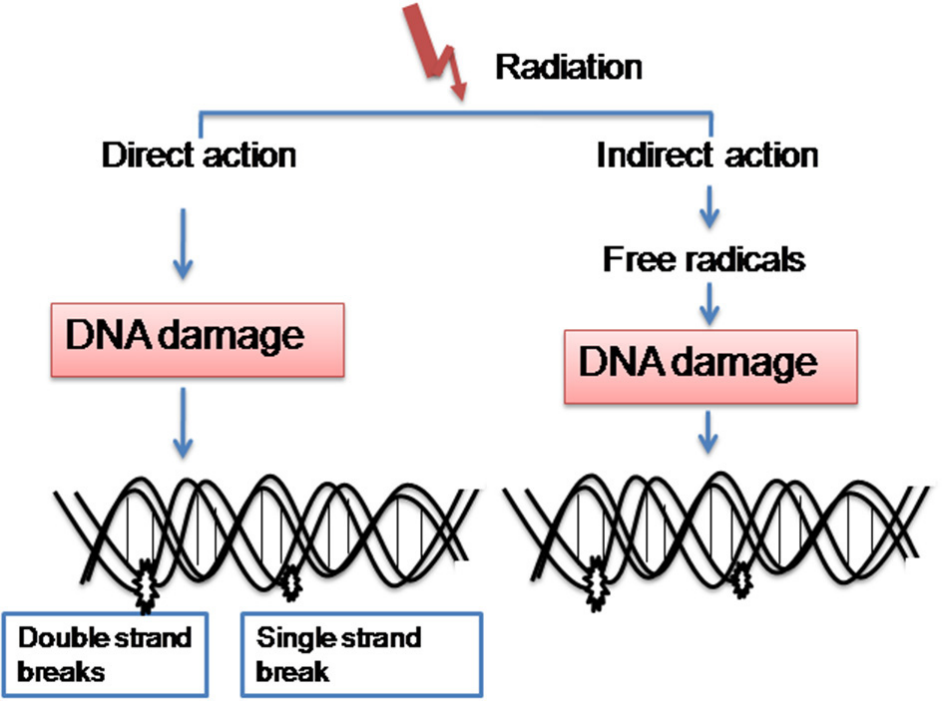
**Radiation mainly acts in two ways**. (1) Induces ionizations directly on the cellular molecules and cause damage. (2) Also acts indirectly, producing free radicals which are derived from the ionization or excitation of the water component of the cells.

Ionizing radiation has been used for more than a century to treat the cancer based on the rationale that the rapidly proliferating cancer cells are sensitive to the radiation treatment than normal cells (Bernier et al., 2004). Under the target-cell damage, the major effect of ionizing radiation on tissues are the direct cell killing mostly by damaging the DNA, resulting in the depopulation of cell populations and subsequent functional deficiency. Radiation induced ionizations can act directly on the cellular molecules and cause damage (Figure 1). Also can act indirectly, producing free radicals which are derived from the ionization or excitation of the water component (80% of a cell is composed of water) of the cells (Figure 1). For ionizing radiations such as low LET X-rays and gamma-rays, 60% of cellular damage is caused by the indirect effects (Barcellos-Hoff et al., 2005). Radiation induced double strand breaks (DSBs) represent the most lethal types of DNA damage, leading to cell death, if unrepaired. However, DNA damage response mechanisms represent a vital line of defense against exogenous and endogenous damage caused by radiation and promote two distinct outcomes: survival and the maintenance of genomic stability.

**Figure 2:**
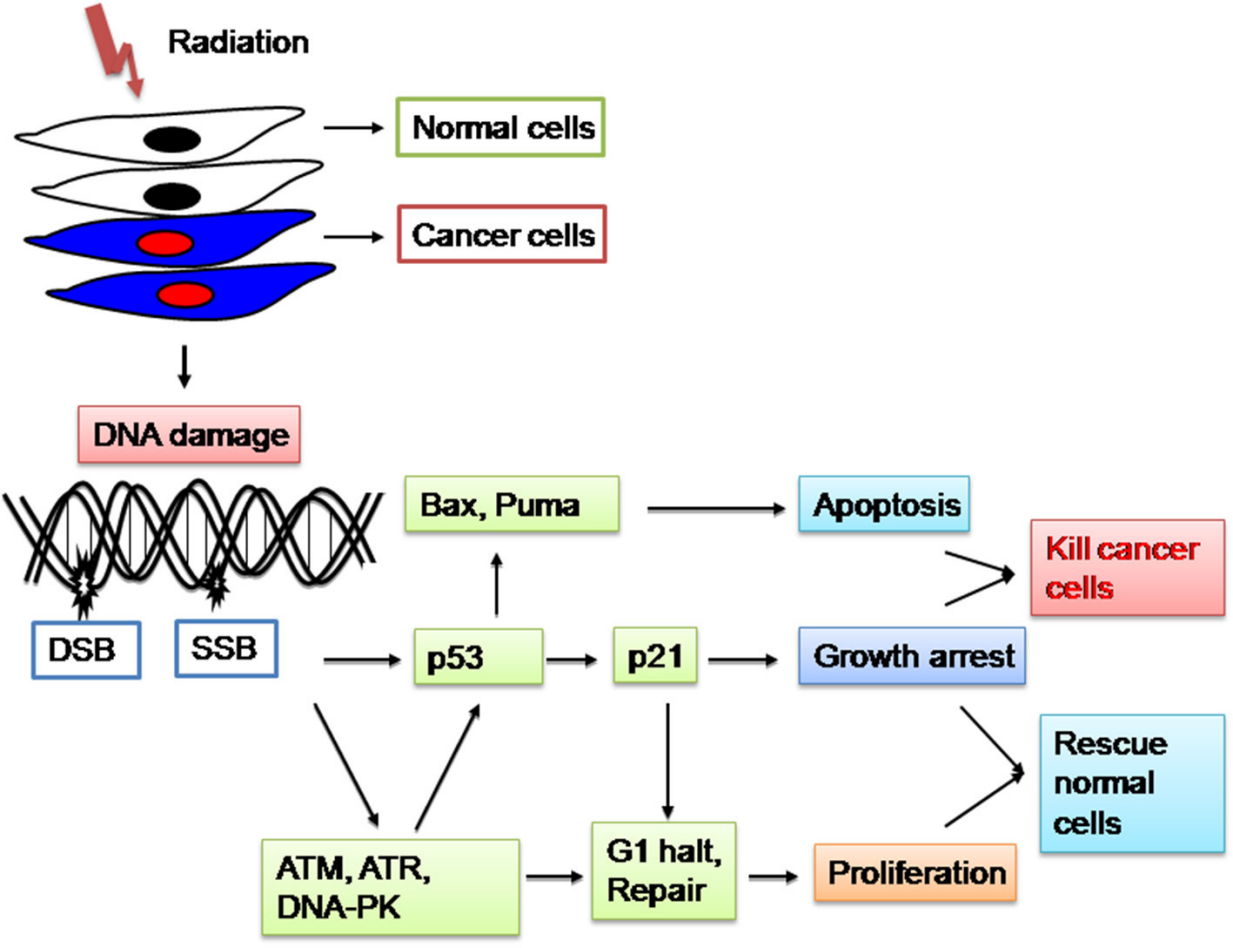
**Radiation damages the genetic material (DNA) causing single strand breaks (SSB) or double strand breaks (DSB) in the cells, thus blocking their ability to divide and proliferate further**. Mechanisms involved in the decrease of radiosensitivity of the fast doubling cancer cells, while increasing radioresistant of the slow doubling normal cells benefits the cancer patients.

Multiple pathways are involved in the genome maintenance of a cell after its exposure to ionizing radiation. Radiation therapy like the most anticancer treatments achieves its therapeutic effect by inducing DNA damage and thereafter cell death (Baskar et al., 2008). Several experiments were performed indicating that the DNA of cancer cells repair more slowly and also produce more DNA breaks (single strand break and double strand breaks) than the normal cells (Parshad et al., 1993; Shahidi et al., 2007, 2010; Mohseni-Meybodi et al., 2009). Furthermore, various proteins involved in cell death and DNA damage mechanisms (Jorgensen, 2009) decrease the radioresistance of the fast doubling cancer cells, while increase in radioresistance of slow doubling normal cells (Figure 2). Therefore, ionizing radiation as applied in the cancer treatment induces a complex response in the cells. Some processes aim to repair the radiation induced damage of the normal cells, whereas others counteract the damage or induce cancer cell death. Growing evidence suggests that various signaling pathways including the DNA repair response pathways shows redundancy in normal cells (Moding et al., 2013). Since cancer cells have various mutations that cause the loss of this redundancy and therefore targeting the DNA damage response pathways in the cancer cells can induce cell death. Hence DNA is the main target for radiation-induced cell killing (Jorgensen, 2009) and there is considerable redundancy in the ability of normal cells to repair DNA damage (Núñez et al., 1996), therefore targeting DNA damage response pathways is a promising approach for the selective radiosensitization of cancer cells (Helleday et al., 2008).

p53 is a transcription factor and also one of the most commonly mutated genes in cancer (Brosh and Rotter, 2009) responds to ionizing radiation by initiating cell cycle arrest, senescence, apoptosis and DNA damage repair (Stiewe, 2007). However, whether p53 induces apoptosis or cell cycle arrest for the DNA damage repair is a complex process and partly depends on the abundance of the p53 protein (low protein levels lead to cell cycle arrest and high protein levels lead to apoptosis) (Lai et al., 2007). However, various DNA repair mechanisms within the tumor cells interfere with the radiation induced damage and further increase the radioresistance of cancer cells (Jorgensen, 2009). Furthermore, inhibition of DNA repair proteins such as ATM or DNA-dependent protein kinase (DNA-PK) have been shown to sensitize the cancer cells to radiation treatment (Veuger et al., 2003; Hickson et al., 2004; Rainey et al., 2008).

Besides the DNA repair pathways, ionizing radiation also triggers cancer cells adaptive cellular responses. Various treatment resistant signal transduction pathways are activated and the resistance can be either intrinsic or an acquired resistance during the fractionated radiation treatment (Toulany and Rodemann, 2013). Signaling pathways that provide cancer cells with a proliferative advantage or allow them to evade the cell death remains a major clinical problem. One of the molecular events by which tumors can become radioresistant is through the ligand-independent activation of signal transduction pathways such as those regulated by membrane-bound receptor tyrosine kinases (RTKs). In this context, epidermal growth factor receptor (EGFR) plays a major role in regulating various downstream signaling pathways, such as the phosphatidylinositol 3-kinase (PI3K) and its downstream kinases such as AKT and mammalian target of rapamycin (mTOR), signal transducer and activator of transcription (STAT) pathway and Ras-mitogen-activated protein kinase (MAPK) pathway (Rodemann et al., 2007; Rodemann and Blaese, 2007). These pathways control the most hallmarks of cancer, including cell cycle, survival, metabolism, invasion, angiogenesis, and genomic instability (Datta et al., 1997; Huang and Harari, 2000; Nyati et al., 2006). Among the prosurvival pathways activated by RTKs, PI3K-AKT-mTOR signaling pathway is frequently upregulated in human tumors and regarded as one of the most challenging prosurvival pathways involved in the resistance to cancer treatment (Engelman, 2009; Liu et al., 2010; Castellano and Downward, 2011).

Recent advances in cancer biology have demonstrated that PI3K-AKT-mTOR signaling pathway controls Fanconi anemia group D2 protein (FANCD2) and ribonucleotide reductase (RNR) and further prolongation of radiation-induced gamma H2AX foci formation (Choi et al., 2010; Shen et al., 2013; Wang et al., 2014). Regulation of DNA repair genes (FANCD2 and RNR) suggests that the PI3K-AKT-mTOR signaling promotes cancer cell survival and resistance to radiation treatment by enhancing the DNA damage repair of the cancer cells. In addition, PI3K-AKT-mTOR signaling pathway also may play a role in the integral functions for non-cancerous (normal) cells repopulation along with the proteins involved in DNA repair mechanisms (Wullschleger et al., 2006). Ionizing radiation also activates NF-κ B transcriptional pathway, through the activation of IκB kinase-α as a protective response to damage and inhibition of this kinase can lead to increased radiosensitvity for the cancer treatment (Brach et al., 1991; Criswell et al., 2003). However, how these pathways inhibition may improve the radiation therapy efficacy in patients remains elusive and the mechanisms underlying the initiation/manifestation of radiation-induced genomic instability in normal and cancer cells are far understood (Bensimon et al., 2013). Furthermore, improvement in preclinical methods for the biological mechanisms involved in signaling pathway(s) for the treatment resistance, cell cycle checkpoints, DNA damage and repair, anti-angiogenesis could increase the therapeutic response of tumor microenvironment, while sparing the surrounding normal tissues. As a result, inhibition of the cancer cells prosurvival pathways has the potential to increase the radiosensitivity of cancer cells through activating/inhibiting multiple mechanisms. Furthermore, inhibition of the cancer cell survival could also affect the radiosensitivity of normal tissues as well, thus decreasing the overall therapeutic index of radiation. Therefore, strategies to improve radiation therapy to increase the effect on tumor while less toxicity on the normal tissues should be achieved without sensitizing the normal tissues and also without protecting the tumors to the radiation treatment.

## Bystander effects

**Figure 3:**
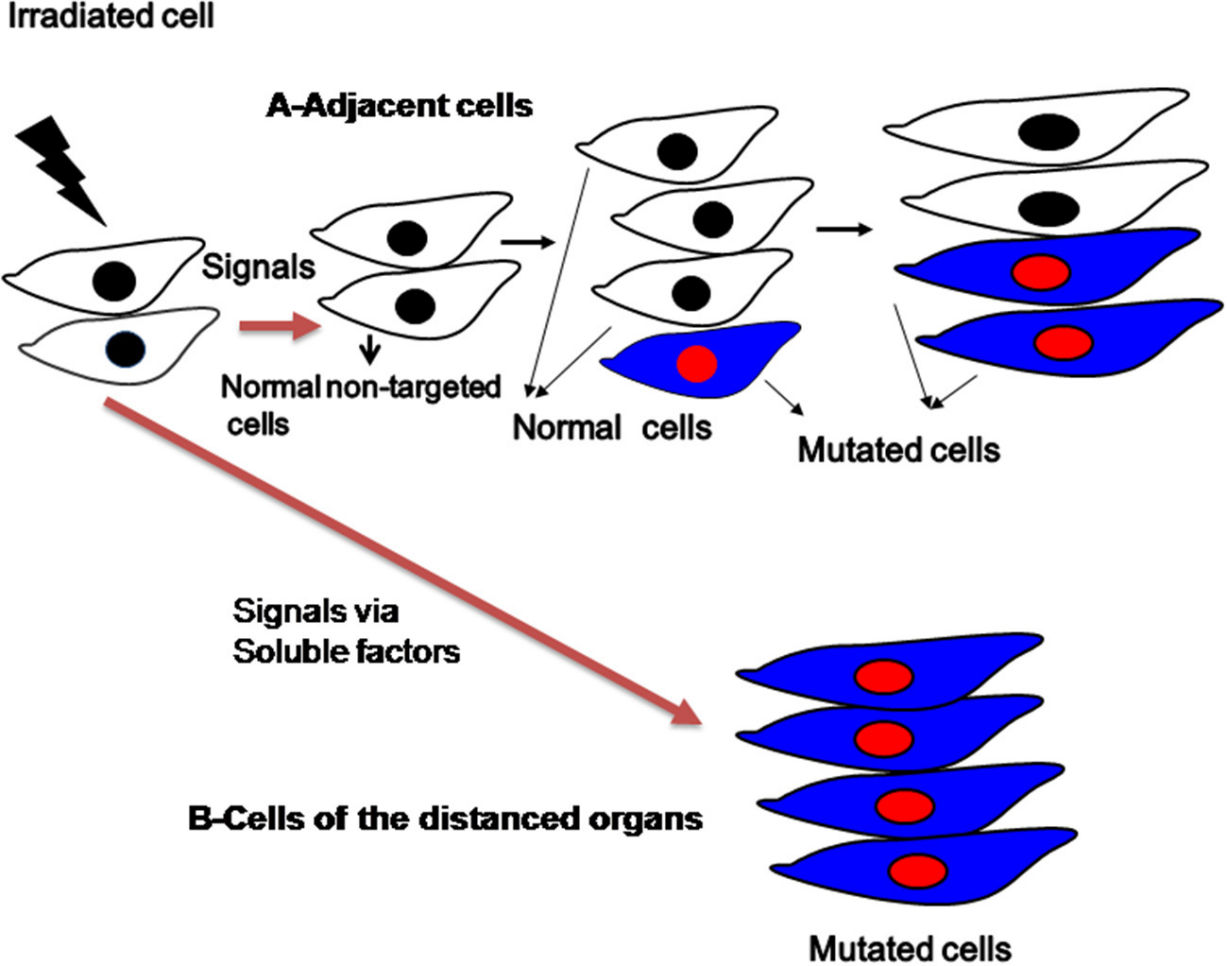
**Schematic representation of bystander effects induced by radiation to the adjacent cells and distanced organs**.

Cancer therapy usually involves exposing the body to agents that kill cancer cells more efficiently than the normal cells. Recent advances in radiation biology and oncology have demonstrated that the radiation is an effective tool to control the localized tumors. However, in recent years mounting evidence indicates that the radiation also can damage not only the cells adjacent to the tumor, but also far from the radiation track by the generation of gap-junction or cytokine-mediated cellular toxicity and also various cellular and microenvironmental signaling cascades are involved (Figure 3) (Shao et al., 2004; Barcellos-Hoff et al., 2005; Baskar, 2010; Butterworth et al., 2013; Suzuki and Yamashita, 2014). In the past two decades, evidence has been mounted for a novel biological phenomenon termed as “bystander effect” (BE). Ionizing radiation induces DNA damage in the form chromosomal aberrations were first reported not only in the directly exposed cells but also in their neighboring non-irradiated cells, termed as radiation-induced bystander effect (RIBE) (Nagasawa and Little, 1992). Therefore, the discovery of non-targeted responses to radiation, such as the bystander response, has called the direct radiation effect paradigm into question. Various biological effects of ionizing radiation are not restricted to only the directly irradiated cells (targeted effects), but are also observed in the progeny of non-irradiated cells (non-targeted effects) (Bensimon et al., 2013). RIBE has been demonstrated in numerous *in vitro* and *in vivo* studies using a variety of biological endpoints. These effects include various molecular and genomic instabilities as seen in the targeted cells. Bystander effects has been extensively studied in the past two decades and reported cell death (Seymour and Mothersill, 1997), induction of sister chromatid exchanges (Nagasawa and Little, 1992; Deshpande et al., 1996), formation of micronuclei (Shao et al., 2003; Balajee et al., 2004; Ponnaiya et al., 2004), mutations (Zhou et al., 2000), delay in cell cycle (Ponnaiya et al., 2004) and transformation (Sawant et al., 2001) of non-irradiated cells along with the proteins involved in the cell cycle and DNA damage response (Hickman et al., 1994; Azzam et al., 1998, 2001; Sokolov et al., 2007; Baskar et al., 2008).

Radiation can cause chromosomal aberrations arising *de novo* in the cell progeny, several generations after irradiation. Delayed genomic instability has been observed in many types of mammalian cells (Ponnaiya et al., 1997; Suzuki et al., 2003; Mothersill et al., 2006; Sudo et al., 2008). Therefore, communication between cells and their microenvironment is critical for both normal tissue homeostasis and tumor growth. RIBE has important implication in tumor control and in radiation therapy, wherein the targeted (directly irradiated) cells transmit the damaging signals to the non-irradiated normal cells, thereby inducing a response similar to that of directly irradiated cells (Mothersill and Seymour, 1998; Shao et al., 2002; Baskar et al., 2007; Baskar, 2010; He et al., 2012). Two major mechanisms mediate RIBE. In the normal and certain cancer cells, mechanisms between cell to cell communications are through the direct gap junction-mediated intercellular communication (adjacent cells/confluent cells) (Azzam et al., 1998; Zhou et al., 2000). Secondly, a range of soluble signaling molecules such as cytokines are involved in the communications between the targeted to distanced non-targeted organs/sub-confluent cells were reported (Ivanov et al., 2010; Hei et al., 2011; Klammer et al., 2013). Among cytokines, tumor growth factor-beta-1 (TGF-β1) has been found to be an important mediator in the bystander effects (Gow et al., 2010; Temme and Bauer, 2013). Recently, Jiang et al. (2014) showed in the lung cancer cells, that the RIBE is mediated by the TGF-β 1–miR-21–ROS pathway. In recent years, number of candidate mediators in bystander effects were identified, among them transforming growth factor-b (TGF-β) (Iyer et al., 2000), tumor necrosis factor-alpha (a) (TNF-α) (Shareef et al., 2007), interleukin-6 (IL-6) (Chou et al., 2007), interleukin-8 (IL-8) (Facoetti et al., 2006) and increase in reactive oxygen species (ROS) (Lyng et al., 2002). RIBE has an important implication in radiation therapy and its impact in radiation oncology is gradually beginning (Munro, 2009). In cancer cells multiple RIBEs, including cell growth stimulation, DNA damage, and cell death have been observed (Sokolov and Neumann, 2010; Veldwijk et al., 2014). However, RIBE is not seen in the human embryonic stem cells (hESC) (Sokolov and Neumann, 2010), indicating stem cells are less susceptible to RIBE than the somatic differentiated cells.

RIBE is also reported using mouse model, the bystander responses of internal tumor cells or tissues were also confirmed *in vivo*, further cancer-associated events such as p53 alteration, MMPs (Matrix metalloproteinases) activity and epigenetic changes were reported in the RIBE (Camphausen et al., 2003; Koturbash et al., 2007). BE can be mediated through an increase in genomic instability, cell cycle delay, cell death (apoptosis), formation of micronucleus, mutations, changes in proteins (gene) expression, and further by malignant transformation (Nagasawa and Little, 1992; Hickman et al., 1994; Shao et al., 2003; Ponnaiya et al., 2004; Baskar et al., 2008). However, the components released from the irradiated cells and further the communication signals involved between the irradiated and non-irradiated cells are still not well known. Recently, Bensimon et al. (2013) showed for the first time in breast cancer cells, a cancer stem cell (CSC) marker CD24 is associated with the transmission of genomic instability of the bystander cells. Recently Aravindan et al. (2014) reported that the clinical doses of abdominal irradiation (2Gy) in mice showed an increase in the onset of NF-kB signal transduction and subsequent NF-kB activation in the non-targeted distant organ (heart). However, little is known about the type of DNA damage of the bystander cells, its radiation resistance and further damage of non-targeted normal cells contributing to tumorigenesis and how this damage can be repaired by designing novel therapeutic approaches to cancer treatment paves a way for an effective strategy to compact the disease.

## Future perspective

Though tremendous progress has been made toward understanding the hallmarks of cancer, cancer is responsible for one in eight deaths worldwide (Garcia et al., 2007; Center et al., 2011). Despite the use of chemotherapy, radiation therapy and surgery, the overall outcome for cancer cure continues to be disappointing. Radiation therapy offers an effective treatment for advanced cancer and the prime goal of radiation treatment is to inhibit the cancer cells multiplication potential and eventually kill the cells. However, radioresistance and repopulation (relapse or recurrence) at the primary site and/or at the malignant areas remain a significant clinical challenge in cancer control. Certain tumors are intrinsically radioresistant, while others acquire radioresistance during the treatment (Seiwert et al., 2007). To overcome the tumor cell radioresistance, it will be a challenging one to identify tumor specific pathways and inhibitors. In the past few years, enormous progress has been made in radiation therapy leading to the possibility of depositing more radiation energy (proton beam radiation therapy, e.g., Bragg Peak) on the tumors while sparing the surrounding normal tissues (Bhide and Nutting, 2010). We do not have a comprehensive answer about the molecular mechanisms involved in the initiation of cancer, developing resistance to treatment and further individual variations in treatment susceptibility, especially of therapy-related beneficial or detrimental effects. In a microenvironment, cancer cells are influenced by various growth signaling pathways to resist the radiation effects and further modify the adjacent normal tissues to impede tumor recurrence or metastasis. Overall, small increase in radioresistance will lead to a large number of cancer cell survivals and further the proliferation forms cancer mass and with a logarithmic decrease in cancer cell death after radiation treatment. Therefore, in the coming years more thrust should be given on the cancer cells radioresistance, e.g., cancer stem cell's radiosensitivity will focus on several different areas along with molecular targeted drugs to control this rapidly growing disease worldwide. Furthermore, with a greater understanding of the tumor biology, evolution of radiation therapy will continue with the improvements in imaging, computing and engineering advancements, and potentially decimate the cancer cells with fewer side effects.

### Conflict of interest statement

The authors declare that the research was conducted in the absence of any commercial or financial relationships that could be construed as a potential conflict of interest.
